# Rethinking Aging: Does an Aging-related Course Influence Student Perceptions of Older Adults?

**DOI:** 10.1093/geroni/igaf122.2953

**Published:** 2025-12-31

**Authors:** Lisa Wagner, Suniska Patel, Courtney Muchow, Elizabeth Hough, Bhumi Jagadish Kotian

**Affiliations:** University of San Francisco, San Francisco, California, United States; University of San Francisco, San Francisco, California, United States; University of San Francisco, San Francisco, California, United States; University of San Francisco, San Francisco, California, United States; University of San Francisco, San Francisco, California, United States

## Abstract

Today’s college students are living through a societal age transformation: older adults were 12.4% of the population when they were born (Administration on Aging, 2006) and will be 23% of the population when they reach older adulthood themselves (Mather & Kilduff, 2020). Yet, many college students have limited experience with older people and little knowledge about aging. In addition to increasing students’ knowledge about aging, aging-related college courses may improve attitudes toward older people. Using a pre-test/post-test design, we assessed attitudes toward older people in college students enrolled in aging-related and non-aging-related courses to determine whether attitudes toward older adults changed following the courses. We hypothesized that attitudes toward older people would improve in Adulthood & Aging and would remain unchanged in non-aging-related courses (Social Psychology, Psychology of Prejudice). We measured attitudes using an allophilia scale (liking for another) modified to measure attitudes toward older adults in five domains: affection, comfort, kinship, engagement, enthusiasm (Wagner & Luger, 2017). Students in Adulthood and Aging had significant increases in comfort, F(1, 58) = 6.8, p = .012), and enthusiasm, F(1, 58) = 8.86, p = .004) for older adults compared to students in non-aging-related courses. Students’ increase in affection F(1, 58) = 3.05, p = .086) was marginally significant. Adulthood and Aging student ratings of kinship and engagement with older adults were significantly higher than non-aging-related courses, F(1, 58) = 9.95, p = .003) and F(1, 58) = 21.1, p < .001, respectively, but the increase over time was not significantly different.

